# Root canal widths of mandibular molars in predicting the legal age threshold 18 years in a sample of juveniles and sub-adults of south-indian origin: an orthopantomographic study

**DOI:** 10.1007/s00414-024-03300-5

**Published:** 2024-07-26

**Authors:** Priyanka Vedula, Ashalata Gannepalli, Sudheer Babu Balla, Gayathri Ch, Anjum Bushra, Bhargavi Krishna Ayinampudi

**Affiliations:** 1 Department of Oral Pathology, Panineeya Mahavidyalaya Institute of Dental Sciences, Hyderabad, India; 2 La Trobe Rural Health School, La Trobe University, Bendigo, Australia

**Keywords:** Dental age assessment, Mandibular molars, Root canal widths, 18 years

## Abstract

Pursuing a proficient age estimation methodology introducing novel radiographic methods remains an ongoing and demanding aspect of forensic and medicolegal research. In 2017, Roberts GJ et al. (J Forensic Sci 62(2):351-4, 2017) described a new radiographic method, i.e., root canal width (RCW) patterns to assign a subject to above 18-year-age threshold. Since then, few researchers have investigated the validity of this radiographic method in other populations. The present study aimed to test the usefulness of these RCW patterns in predicting 18 years in a sample of South Indian juveniles and sub-adults aged between 16 and 23. Descriptive analysis revealed that pattern-A was initially observed at a minimum age of 16.08 and 16.22 years in males and females. Pattern-B at 16.31 years in males and 16.22 years in females, while pattern-C was initially recorded at 18.73 years in males and 19.01 years in females, respectively. In summary, if an individual, regardless of sex, exhibits a fully-formed (apex closed) mandibular first, second, and third molars and concurrently displays RCW-C, there is a strong likelihood that the person has exceeded the legally relevant age of 18 years. However, due to higher rate of technically unacceptable errors (adults wrongly identified as individuals below 18 years), reliance on this method alone should be restricted, and it is advisable to combine it with other methods.

## Introduction

Determining the age of juveniles and sub-adults is a common practice in various legal contexts, such as criminal investigations, civil cases, and assessments of refugee migrant populations [1]. Accurate age assessment is crucial, given that legal provisions often hinge on an individual’s age. In many countries, evidence of falling within or exceeding legally stipulated age thresholds is necessary for legal determinations regarding procedural rights or social entitlements [2]. In many jurisdictions, reaching 18 years signifies attaining legal adulthood, with this age serving as a prominent demarcation in criminal and civil proceedings [3]. At 18, individuals are considered adults, becoming accountable for their decisions and actions and assuming legal control [4]. Misclassifying an individual’s age beyond the legal threshold of 18 carries substantial legal and ethical consequences, as treating a minor erroneously as an adult can lead to severe repercussions [5, 6]. Therefore, employing the least invasive procedures and a combination of age estimation methods is imperative to ensure the highest accuracy and minimise the risk of misclassification [7].

As per the recommendations by the Study Group on Forensic Age Diagnostics, age assessments conducted for criminal proceedings should encompass a thorough physical examination, scrutiny of sexual maturation, an X-ray of the left hand, and a dental examination that documents dentition status and include an evaluation through orthopantomography. Furthermore, it is recommended to undergo a radiological or CT examination of the clavicle when the skeletal maturation of the hand is complete [7]. The assessment involves evaluating the degree of dental development, dental eruption, maturation of hand and wrist bones, long bone growth, and epiphyseal fusion. These anatomical structures serve as key indicators of biological maturity [8]. Among them, dental development exhibits a stronger correlation with chronological age. It appears to be significantly influenced by genetic factors, while bone development is more susceptible to external factors like nutrition and overall health [8, 9].

To address the pertinent legal question of whether an individual has reached 18 years of age, assessing the mineralisation of third molars becomes crucial, as all other teeth have typically completed their maturation by this stage. Various methods exist for predicting the attainment of the legal age threshold of 18 years, including Mincer et al. stages (specifically stage H) [10], the third molar maturity index proposed by Cameriere et al., [11] wisdom tooth eruption as suggested by Olze et al., [12] and the stages of root pulp visibility (RPV) and periodontal ligament visibility (PLV) proposed by Olze et al. [13, 14]. Various theories attempt to explain the reduced visibility of root canals or pulp. One theory attributes it to an optical phenomenon [13]. While the exact physiological mechanism remains unclear, secondary dentin formation in the pulp likely contributes to this effect [15]. Additionally, changes in bone or tooth structure, such as the densification of dentin and decrease in dentinal tubule diameter near the pulp over time, may impact radiopacity and the representation of fine root pulp radiologically [16]. Recognizing this phenomenon, Roberts et al. identified a new growth marker based on the relative width of the distal root canals of the lower left permanent molars (36, 37, and 38 in FDI dental notation) as visualised on dental panoramic radiographs [17]. Their findings indicated that stage C of root canal width (RCW) in females and RCW-B & C in males are compelling evidence that a subject is older than 18. However, it is noteworthy that, as of now, very few have validated the reliability of Roberts et al.‘s method concerning the relative width of the root canals of lower molars [18–20]. Consequently, we aim to test whether Robert et al. RCW patterns of lower molars can be considered a reliable indicator for identifying individuals over 18 in a sample of south-Indian juveniles and sub-adults.

## Materials and methods

### Sample

A total of 924 digital orthopantomograms (OPGs) from individuals of South Indian origin, aged between 16 and 23 years, were retrospectively gathered from the digital archives of the Department of Radiology at Panineeya Institute of Dental Sciences and private clinics. The distribution of the total sample according to age and sex is presented in Table 1. Ethical approval was secured from the institutional ethics committee before sample collection (PMVIDS&RC/IEC/OMFP/PR/0285 − 18). Details such as the patient’s date of birth, the date of radiograph exposure, and information regarding the individual’s sex were considered in the study. All radiographs were assigned anonymous numbers before evaluation, ensuring randomisation of study participants and masking participant details, including age and sex, from observers during the assessment. The chronological age of each individual was calculated in decimal form by subtracting the date of birth from the date of radiograph exposure.

**Table 1:** Age and sex-wise distribution of the total sample

Age groups	Males	Females	Total
16-16.99	64	68	132
17-17.99	62	64	126
18-18.99	64	66	130
19-19.99	72	60	132
20-20.99	72	68	140
21-21.99	72	72	144
22-22.99	60	60	120
Total	466	458	924

The inclusion criteria comprised OPGs demonstrating good image quality, specifically showcasing the intact right or left set of all three mandibular molars with fully formed roots, denoted as stage H in the eight-stage system of Demirjian’s tooth development stages [21]. Molars exhibiting incomplete root formation, signs of caries, dental fillings, evidence of endodontic treatment, poor image quality, or compromised integrity of root pulp were excluded from the study. The assessments were consistently conducted on the left molars, with right molars considered for evaluation only in cases where assessment of the left molars was not feasible. The study was carried out under the ethical standards laid down by the Declaration of Helsinki (Finland) and its later amendments [22].

### Method

The assessment of relative widths of the distal root canals of the lower left first, second, and third molars was recorded, as defined by Roberts GJ et al. [17], in the following three stages, RCW-A, B, and C.

**Fig. 1:**
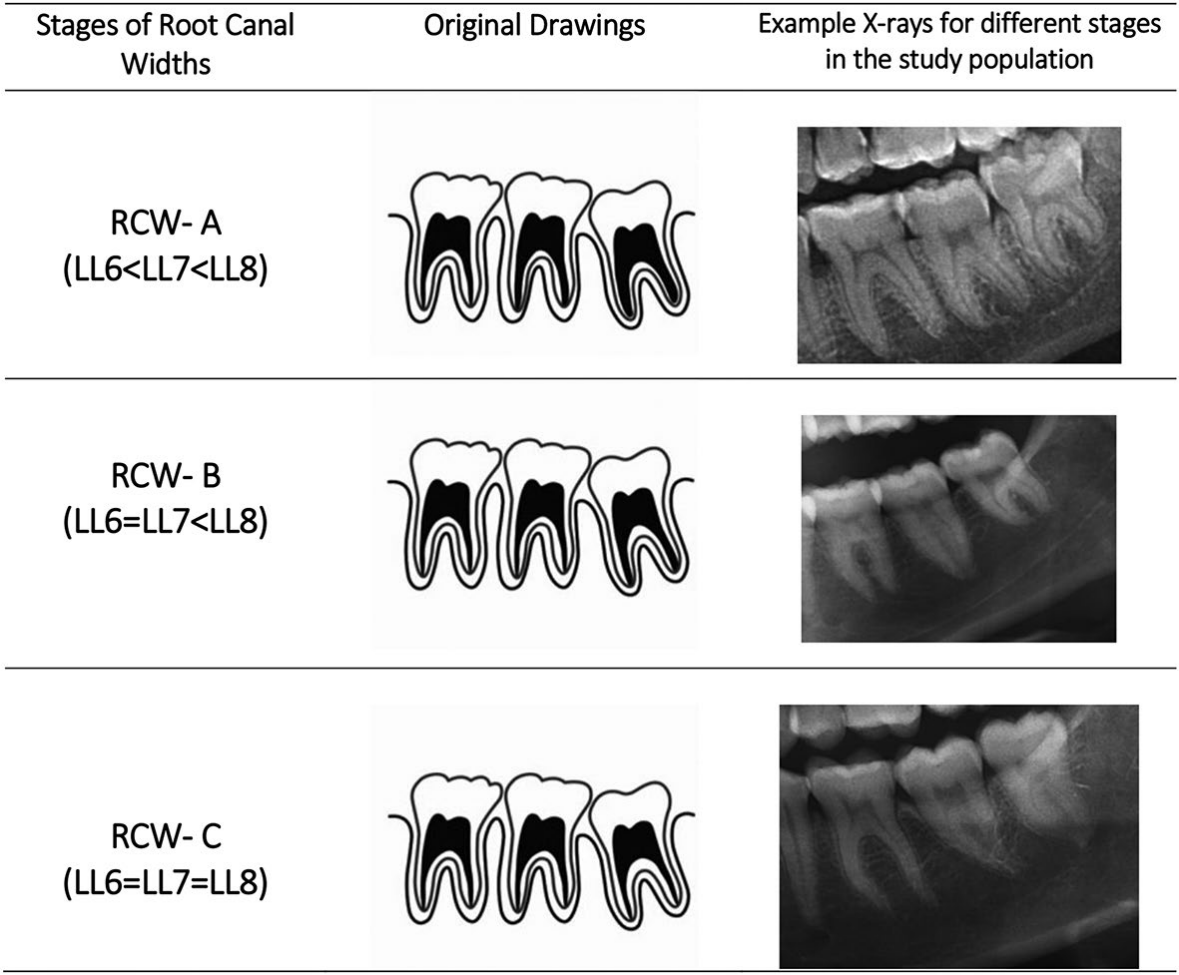
Schematic representations of the three categories of root canal width (RCW) proposed by Roberts GJ et al., 2016 [17]

In RCW-A, the relative mesiodistal width of the distal root canal in the lower left first molar is narrower than the relative mesiodistal width of the distal root canal in the lower left second molar, which in turn is narrower than the relative mesiodistal width of the distal root canal in the lower left third molar. In RCW-B, the relative mesiodistal width of the distal root canal in the lower left first molar is equal to the relative mesiodistal width of the distal root canal in the lower left second molar, which in turn is narrower than the relative mesiodistal width of the distal root canal in the lower left third molar. And, in RCW-C, the relative mesiodistal width of the distal root canal in the lower left first molar is equal to the relative mesiodistal width of the distal root canal in the lower left second molar, which in turn is equal to the relative mesiodistal width of the distal root canal in a lower left third molar (Fig. 1). Those root canal patterns that did not fall into the categories as described by Roberts GJ et al. [17] was recorded under a new category, “RCW-U,” as proposed by Davidson et al. [18].

A forensic odontologist (SBalla) with ten years of experience in forensic age estimation evaluated all the orthopantomograms (OPGs). To gauge the method’s reliability, a hundred OPGs were randomly chosen and examined by two authors to assess inter-observer variability. The second observer (PV) is a specialist in dentistry with three years of experience in forensic age estimation. Additionally, SBalla independently assessed another fifty randomly selected OPGs twice, with a three-month interval between the assessments, to evaluate intra-observer variability.

### Statistical analysis

Cohen’s kappa statistics were employed to determine both intra- and inter-observer agreement. A descriptive analysis of the relative distal RCW patterns in lower molars was conducted based on chronological age. The relationship between age and pattern attainment was explored using a Chi-square test. The correlation between chronological age and the RCW pattern was performed using Spearman rank order correlation (rho).

To assess the viability of RCW-C as an age marker for the 18-year age threshold, 2 × 2 contingency tables were generated. These tables illustrated the counts of true positives (individuals attaining RCW-C and older than 18), false positives (individuals attaining RCW-C and younger than 18), true negatives (individuals not attaining RCW-C and younger than 18), and false negatives (individuals not attaining RCW-C and older than 18 years). The evaluation included measurements of accurately classified subjects, the sensitivity of the test (Se), indicating the proportion of subjects who are truly above the age threshold of interest, and the specificity of the test (Sp), indicating the proportion of subjects who are truly below the age threshold of interest.

Data registration was carried out using Microsoft Excel (Microsoft Corp., Redmond, WA, USA), and statistical analyses were conducted using IBM SPSS Statistics 29 (SPSS Inc., Chicago, IL, USA). The level of significance for the analyses was set at *p* < 0.05.

## Results

Cohen’s kappa statistics reveal high reproducibility and repeatability within the same observer, with an intra-observer agreement value of 0.754. In contrast, the inter-observer agreement is indicated by a value of 0.671, signifying a substantial level of agreement.

Table 2 illustrates the outcomes of the descriptive analysis, presenting the means, standard deviations (SD), minimum age, first quartile, median, third quartile, and maximum age of subjects for both males and females in each RCW pattern. The mean chronological age for RCW-A was 18.01 and 17.67 years in males and females, respectively. For RCW-B, it was 19.11 and 18.52 years, while for RCW-C, it was 21.29 and 21.41 years in males and females, respectively. The minimum age values for stages RCW-A and RCW-B were observed to fall below the 18-year-old threshold for both sexes, while the earliest appearance of RCW-C was found to be above the 18-year threshold for both males and females.

**Table 2:** Summary data for each of the grades of root canal widths for males and females

Grade	*N*	Mean	SD	Min	25th	50th	75th	Max
**Males**								
RCW-A	168	18.01	1.36	16.08	16.98	17.7	19.07	20.76
RCW-B	84	19.11	1.49	16.31	17.95	18.87	19.86	22.38
RCW-C	168	21.29	1.11	18.73	20.64	21.43	22.31	22.94
RCW-U	46	20.74	1.64	17.13	20	21.06	22.24	22.55
**Females**								
RCW-A	106	17.67	1.15	16.22	16.76	17.28	18.47	19.93
RCW-B	138	18.52	1.36	16.22	17.63	18.36	19.23	21.99
RCW-C	176	21.41	1.04	19.01	20.43	21.76	22.34	22.94
RCW-U	38	20.39	1.95	17.45	18.25	21.22	22.08	22.82

RCW Root canal width; N number; SD Standard Deviation; Min Minimum; Max Maximum

Table 3 demonstrates the sample distribution by sex and age group, i.e., above or equal to and below 18 years. It was observed that RCW-C was attained by males and females (100%) older than 18. Spearman rho correlation found that there was a positive correlation between the variables for both sexes (Spearman rho = 0.693, *p* < 0.05 and Spearman rho = 0.707, *p* < 0.05, for males and females, respectively).

**Table 3:** Distribution of the sample (percentage), by sex and age group, according to the grade of root canal widths

Sex	Age group	Grade of root canal widths			
		RCW- A	RCW- B	RCW- C	RCW- U
Males	< 18 years	92 (54.8)	28 (33.3)	0 (0.0)	4 (8.7)
	*≥* 18 years	76 (45.2)	56 (66.7)	168 (100)	42 (91.3)
Females	< 18 years	68 (64.2)	52 (37.7)	0 (0.0)	6 (15.8)
	*≥* 18 years	38 (35.8)	86 (62.3)	176 (100)	32 (84.2)

In total, 9.1% (86 out of 924) of the samples were classified under the RCW-U pattern. This pattern consisted of three distinct RCW sub-patterns that did not align with the RCW patterns outlined by Roberts GJ et al. [17] 73.8% of the RW-U patterns was LL6 < LL7 = LL8, followed by LL6 < LL7 > LL8 (16.7%), and LL6 > LL7 = LL8 (9.5%) (Table 4).

**Table 4:** Summary data of the different patterns of RCW-U in both genders

Pattern of RCW-U	Males		Females		Total	
	*n* (%)	Median	*n* (%)	Median	*n* (%)	Median
LL6 < LL7 = LL8	32 (69.5)	20.66	30 (78.9)	21.22	62 (73.8)	21.06
LL6 < LL7 > LL8	8 (17.4)	21.33	6 (15.8)	22.16	14 (16.7)	21.99
LL6 > LL7 = LL8	6 (13.1)	22.24	2 (5.3)	22.31	8 (9.5)	21.31
Total	46 (100)	--	38 (100)	--	84 (100)	--

Table 5 presents the outcomes regarding the efficacy of RCW-C utilising 2 × 2 contingency tables. The overall proportion of accurately classified participants for males was 68.5% (95% CI, 63.8–72.9%). The sensitivity was 56% (95% CI, 50.2–61.7%), and the specificity was 100% (95% CI, 96.9–100%). The overall proportion of accurately classified participants in females was 70.4% (95% CI, 65.8–74.8%). The sensitivity was 58.6% (95% CI, 52.8–64.3%), and the specificity was 100% (95% CI, 96.9–100%). Category RCW-C appeared to suggest that individuals were 18 years and over for both males and females. The probability of individuals being over 18 was 1.00 (100%) in males and females if category RCW-C was present.

**Table 5:** The quantities derived from 2 × 2 contingency table (95% confidence interval) of test of the age of majority in the south Indian juveniles and sub-adults when stage RCW- C was used to discriminate minors or adults in both sexes

Criteria	Males	Females
TP	120 (47.6)	120 (49.2)
TN	168 (100)	176 (100)
FP	0 (0.0)	0 (0.0)
FN	132 (52.4)	124 (50.8)
Sensitivity	56 (50.2–61.7)	58.6 (52.8–64.3)
Specificity	100 (96.9–100)	100 (96.9–100)
Accuracy	68.5 (63.8–72.9)	70.4 (65.8–74.8)

TP True positive; TN True negative; FP False positive; FN False negative

## Discussion

The reliability of tooth development as a human biological growth marker stems from its resistance to influences such as genetics, nutrition, climate, hormones, and the environment. Being largely independent of exogenous factors and seldom affected by pathological conditions further establishes it as a dependable growth indicator in human biology [23–25]. Age estimation, particularly in young adults, primarily relies on the morphological characteristics of bones and the mineralisation of third molars. After age 14, third molars are the sole teeth undergoing developmental processes, making them crucial for forensic age estimation [26, 27]. Despite variations in position, morphology, and development, these teeth remain a focal point, especially for distinguishing between adults and minors. Existing literature supports extensive research on these teeth concerning their development, emergence, calcification, and regressive changes linked to chronological age [10–14]. However, this study, to the best of the authors’ knowledge, represents the first attempt to assess the capability of Robert et al.‘s stages of relative widths of root canals in lower molars [17] for discriminating south Indian juveniles and subadults concerning the age threshold of 18 years.

Robert et al. suggest that the pattern observed in each category of root canal width is founded on the premise that as individuals age increases, the relative mesiodistal widths in the distal roots of all lower three molars decrease [17]. In our study, we noted a trend of root canal narrowing following the closure of the apex in the molars. The findings from this validation study indicated that all males and females in the studied population with stage H left molars and RCW-C pattern were over 18 years old. These results align with the findings reported in the original study [17] and two validated studies conducted on South African and Maltese populations [18, 19]. In males, the most common patterns were RCW-A and RCW-C, each accounting for 40%, while in females, RCW-C was the most prevalent at 42%, followed by RCW-B at 33%. These findings contrast with Tangkabutra et al.‘s report, [19] where they identified RCW-C as the least common pattern. Additionally, the results demonstrated that as the categories increased (RCW-A to C), there was a corresponding rise in the mean chronological age for both males and females. Furthermore, the first (25th percentile), median (50th percentile), and third (75th percentile) quartiles displayed an increase in age from RCW-A to RCW-C. This suggests no significant differences between sexes in RCW development when a specific pattern was assigned.

A Spearman correlation test was used to evaluate the strength and direction of the linear relationship between RCW patterns and chronological age. The results showed a moderate positive correlation for both males and females, meaning that RCW patterns tend to increase with age. However, the moderate correlation is not easily explained. It is likely that the limited number of RCW patterns in the classification does not adequately capture the chronological progression of the characteristic being studied. Various factors influence the accuracy of age estimates, and one crucial aspect is the observer’s performance. The precision of dental age estimation is closely tied to the assessment method employed [28]. Despite the well-established characteristics distinguishing each developmental stage, the classification of tooth developmental stages can be subjective and susceptible to inter- and intra-observer variability [29]. Our study found a satisfactory intra-observer agreement (0.75), but the inter-observer agreement was comparatively lower (0.67). These results exhibited lower inter- and intra-observer agreements compared to the original study, where agreements surpassed a kappa score of 0.9 [17]. However, they align more closely with the outcomes of Davidson et al. [18] and Tangkabutra et al. [19]. The differences observed compared to the original study may be attributed to the greater experience of the original method developers [19]. Similar observations were noted in a study by Timme et al., [20] where they reported poor agreement between observers, i.e., 0.07 (95% CI: −0.11–0.26; 40.7% agreement) for females and 0.11 (95% CI: −0.08 − 0.31; 41.7% agreement) for males, indicating limited consistency. They suggested that the lack of reference points in the method might contribute to poor agreement between observers. Recognising patterns becomes challenging, especially considering the variations in shape and size between the first and second molars compared to the third molars. They further emphasised the need to consider this pattern recognition approach carefully, particularly in light of inter-observer agreement concerns.

In forensic age assessment, experts frequently need to determine the most likely age and/or the minimum age of the individual being examined. In 2016, Schmeling et al. [30] introduced the “minimum age concept,” aimed at preventing the misclassification of minors as adults. This concept is designed to provide a high level of certainty regarding the attainment of the age of majority. However, it often relies on a single observation rather than considering the entire reference sample centered around the mean age of the stage. In this study, we incorporated the minimum age concept to mitigate the risk of age mimicry bias. Our findings suggest that stage RCW-C indicates a minimum age of 18.73 years in males and 19.01 years in females, respectively. These findings are consistent with those of the original study, where the minimum age for RCW-C was reported as 18.16 years for males and 18.45 years for females [17]. Additionally, our results align with the conclusions drawn by Davidson et al. and Tangkabutra et al., emphasising the persistent capability of RCW-C in effectively differentiating between adults and minors [18, 19].

Legal professionals and governmental entities frequently seek the expertise of forensic specialists for age estimation in individuals involved in civil and criminal litigations. However, the necessary probabilities to meet the criteria for age estimation differ depending on the context of the assessment. The requirement is typically at least 90% for criminal proceedings, whereas it is 51% for civil proceedings [30]. In criminal law, there is a stringent demand for age assessment or classification to be provided with a very high probability, with a particular emphasis on minimising false positive rates [31]. Garamendi et al. have distinguished errors in age assessment into two categories: technically unacceptable errors (false negative) and ethically unacceptable errors (false positive). Ethically unacceptable errors, especially in criminal proceedings, are considered the most harmful to the child’s best interest [32]. Therefore, it is crucial to thoroughly investigate and accurately report the false positive rate associated with the applied methods. In this context, stage RCW-C in our current study achieved a specificity of 100% in both males and females. However, our findings indicate that RCW-C exhibits lower sensitivity, suggesting a potential for a high percentage of adults being incorrectly classified as minors in the studied population. Therefore, the conclusion drawn from these findings is that when an individual, whether male or female, presents with fully formed mandibular first, second, and third molars (stage H of Demirjian’s staging system) for age assessment, and stage RCW-C is concurrently observed, there is a strong likelihood that the individual has surpassed the legally relevant age of 18 years. However, it is essential to exercise caution in its application due to inherent limitations.

There are several methodological issues in identifying root canal width (RCW) patterns. The first issue concerns root canal morphology. The initial assessment involves making decisions about the distal root canals, specifically the mesiodistal width of the root pulp in all lower three molars. Singh et al. [33] examined the root canal morphology of the lower three molars in South Indians, finding that 90% of the lower first molars and 91% of the lower second molars were two-rooted, while only 63% of the lower third molars had two roots. This variability might limit the method’s applicability to the studied population. Another issue relates to the original RCW classification. The foundational work by Roberts GJ et al. [17] proposed a distinct classification of RCW stages, illustrated in Fig. 1. However, our systematic analysis revealed discrepancies between these patterns and those depicted by Roberts GJ et al. [17] Subsequently, Davidson et al. [18] introduced an additional category, RCW-U, to accommodate patterns not fitting into the initial three categories. Studies have reported that the prevalence of the RCW-U category ranges from 15.47 to 35.87% [18, 19]. In our study, we found that less than 10% of the samples fell into the RCW-U category. The most common RCW-U pattern we observed was LL6 < LL7 = LL8 (73.8%), which aligns with Davidson et al.‘s finding of 71.43% for this pattern. The reasons behind these varying patterns are unclear but might be due to differences in secondary dentin deposition rates among individuals or variations in panoramic radiographic acquisition, projection geometry, and the focal trough, all of which can affect the image characteristics of the root canal width in third molars [17]. This study has several limitations. Firstly, it focused on a somewhat geographically restricted sample, comprising participants from five South Indian states and excluding other regions in India. Future research could benefit from validating this method in diverse geographic areas, establishing a more representative and comprehensive sample. Secondly, the method’s reliance on the presence of all three molars poses a challenge, given the notably higher prevalence of congenitally missing lower third molars in the studied population [34, 35]. Additionally, the evaluation of the method may be constrained in samples lacking a clear definition of root canals, particularly in impacted lower third molars where visibility is hindered by physiological thickening and compaction of the mandibular bone in the wisdom teeth region [20]. Factors such as agenesis and impaction of third molars could influence the method’s application or the assignment of the RCW pattern, negating its use as a maturity marker. Thirdly, another limitation is the potential for radiographic distortion or alterations in the projection of root canal width onto the film or sensor due to the use of two-dimensional images [36]. The use of cone-beam computed tomography (CBCT) could mitigate this issue, providing more accurate measurements.

Despite these limitations, which are common to many age estimation methods, we believe this approach paves the way for exploring new methods to determine the legal age threshold of 18 years. Additional research employing this methodology is needed across varied populations to evaluate its effectiveness and to compare the findings of this study, thereby strengthening confidence in this approach.

## Conclusion

After analysing a sample of 924 juveniles and sub-adults from south India aged between 16- and 23-years using Robert et al.‘s stage classification, it can be inferred that this method serves as a growth marker effectively indicating individuals over 18 years old in the studied population. The study found that no individual under 18 years of age was mistakenly identified as an adult when the RCW-C pattern was used. However, almost half of the adults were incorrectly identified as being under 18, showing a high rate of significant (technically unacceptable) errors with the RCW-C pattern. Therefore, this method should not be used alone, and it is recommended to combine it with other methods. Additional research is needed to validate the applicability of RCW patterns in diverse populations using three-dimensional radiographic techniques.

:
